# Peripheral blood DNA methylation profiles predict future development of B-cell Non-Hodgkin Lymphoma

**DOI:** 10.1038/s41698-022-00295-3

**Published:** 2022-07-21

**Authors:** Almudena Espín-Pérez, Kevin Brennan, Asiri Saumya Ediriwickrema, Olivier Gevaert, Izidore S. Lossos, Andrew J. Gentles

**Affiliations:** 1 Stanford Center for Biomedical Informatics Research (BMIR), Department of Medicine, Stanford University, Stanford, CA 94035 USA; 2 Division of Hematology, Stanford University School of Medicine, Stanford, CA 94035 USA; 3 Department of Medicine, Division of Hematology, Miller School of Medicine, University of Miami, 1600 NW 10th Avenue/1475 NW 12th Avenue (D8-4), Miami, FL 33136 USA; 4 Sylvester Comprehensive Cancer Center, University of Miami, Miami, FL USA; 5 Department of Molecular and Cellular Pharmacology, University of Miami, Miller School of Medicine, Miami, FL USA; 6 Department of Biomedical Data Science, Stanford University, Stanford, CA 94035 USA; 7 Cancer Institute, Stanford University, Stanford, CA 94035 USA

**Keywords:** Cancer genomics, B-cell lymphoma, High-throughput screening

## Abstract

Lack of accurate methods for early lymphoma detection limits the ability to cure patients. Since patients with Non-Hodgkin lymphomas (NHL) who present with advanced disease have worse outcomes, accurate and sensitive methods for early detection are needed to improve patient care. We developed a DNA methylation-based prediction tool for NHL, based on blood samples collected prospectively from 278 apparently healthy patients who were followed for up to 16 years to monitor for NHL development. A predictive score was developed using machine learning methods in a robust training/validation framework. Our predictive score incorporates CpG DNA methylation at 135 genomic positions, with higher scores predicting higher risk. It was 85% and 78% accurate for identifying patients at risk of developing future NHL, in patients with high or low epigenetic mitotic clock respectively, in a validation cohort. It was also sensitive at detecting active NHL (96.3% accuracy) and healthy status (95.6% accuracy) in additional independent cohorts. Scores optimized for specific NHL subtypes showed significant but lower accuracy for predicting other subtypes. Our score incorporates hyper-methylation of Polycomb and *HOX* genes, which have roles in NHL development, as well as *PAX5* - a master transcriptional regulator of B-cell fate. Subjects with higher risk scores showed higher regulatory T-cells, memory B-cells, but lower naïve T helper lymphocytes fractions in the blood. Future prospective studies will be required to confirm the utility of our signature for managing patients who are at high risk for developing future NHL.

## Introduction

Non-Hodgkin lymphoma (NHL) is a malignancy of the lymphoid system which can often spread to distal organs. If NHL is detected early, patients are more likely to have a favorable outcome since stage I patients have 84% 5 years survival; whereas Stage IV patients have 64% survival^1,2^. NHL is the 7th most prevalent type of cancer in the U.S. According to the National Institutes of Health, approximately 2.2% of men and women will be diagnosed with NHL at some point during their lifetime. It is estimated that 80,470 NHL cases will be diagnosed and 20,250 deaths will occur in 2022 in the US alone. The overall 5-year survival rate is around 70%^1^.

Early treatment intervention is crucial, as it is more difficult to treat patients with advanced disease. Previous studies had observed aberrant global DNA methylation in samples collected close to diagnosis of blood cancers^3^, motivating the research of early molecular changes associated with cancer initiation. Despite significant efforts, the impact of biomarkers for early detection of cancer has remained limited. The International Lymphoma Epidemiology Consortium (InterLymph)^4,5^ aims to identify common as well as distinct risk factors for developing NHL among NHL subtypes. While the Consortium has identified genetic, environmental and demographic risk factors, currently there are no predictive models that can robustly quantify the risk of developing NHL. This limitation is partly due to the heterogeneity of NHL, which is a major challenge for developing such models. While identifying subjects at risk for NHL could aid in early diagnosis, to date there are no recommended screening tests for lymphomas. The typical scenario for early diagnosis of NHL is regular medical check-ups with attention to symptoms and known risk factors for NHL such as first-degree family history of NHL, age and autoimmune disorders. If NHL is suspected of causing patient symptoms, a biopsy of a swollen lymph node or other affected area may be performed.

Detection of altered DNA methylation in liquid biopsies is a promising approach to detect cancer early^6^. Methylation of DNA at genomic CpG sites can regulate gene expression without changing the underlying DNA sequence. Since DNA methylation alterations can reflect genetic background, environmental factors, aging, Epstein-Barr virus, tissue composition, smoking and other lifestyle factors, these alterations could represent surrogate markers of underlying NHL risk factors. The association between aberrant patterns of DNA methylation and cancer development has long been recognized, including in blood malignancies^7,8^. In particular, Polycomb group proteins (histone-modifying enzymes involved in maintaining stem cell identity and orchestrating cellular differentiation during development) play a central role in malignant transformation of lymphomas^9,10^. In addition, DNA methylation is the only epigenetic modification that is stable enough to be measurable in archival tissue samples, allowing the exploitation of existing biobanks by correlating molecular profiles with health outcomes over the course of a lifetime.

Harnessing quantification of genome-wide DNA methylation for developing predictive and prognostic models require mining large volumes of data involving complex interactions. Artificial intelligence algorithms and rigorously-defined statistical learning frameworks have become indispensable tools for biomarker discovery from genomics data generated by high-throughput technologies in population studies^11^, especially for the study of complex diseases like NHL whose origin is not driven by one variable (e.g., a monogenic factor) but rather by many features (genes and gene-environment interactions)^5,12–14^.

**Fig. 1:**
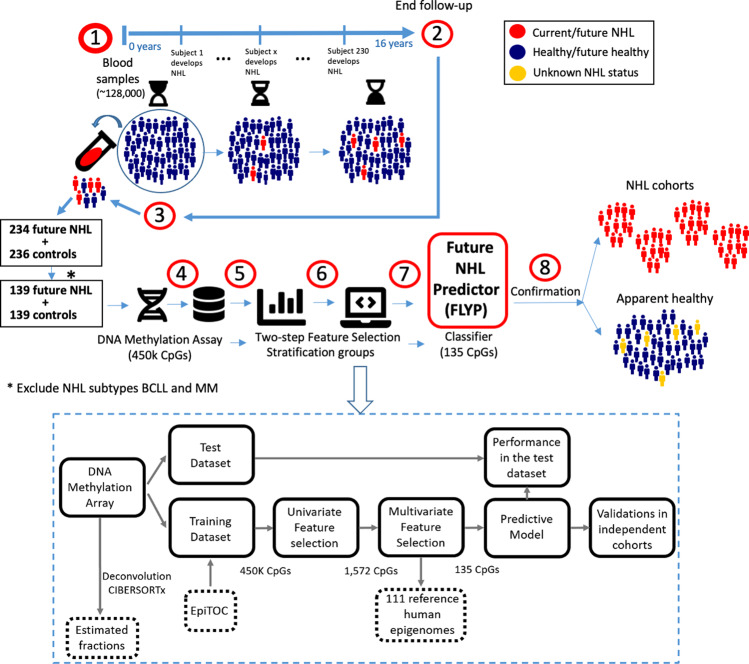
Workflow of the study. Blood samples from a large population of healthy volunteers were taken and frozen using liquid nitrogen (1). Throughout a 16 years follow-up, subjects that developed NHL were registered (2). At the end of the follow-up, a balanced cohort comprising individuals that developed NHL and a matching ratio of controls was identified (3) and omics analyses were conducted on the selected frozen blood samples (4). Data was preprocessed and analysed to develop a classifier for future NHL called Future LYmphoma Predictor or FLYP (5,6,7). Finally, the classifier was tested in a variety of independent NHL and healthy cohorts (8). BCLL = B-cell chronic lymphocytic leukaemia, MM = multiple myeloma.

Here we developed a computational framework to identify features associated with future (lead time up to 16 years) development of NHL, defining a predictive signature based on DNA methylation profiles from blood samples (Fig. 1). DNA methylation information from prospectively gathered samples has potential for developing predictive models but is challenging to collect. We address these limitations by dissecting the complexity underlying NHL prediction using a variety of computational approaches and large-scale population studies, boosting the signal-to-noise ratio of a subset of subjects showing measurable key features of future lymphoma development.

## Results

### Polycomb and HOX gene hyper-methylation is associated with future development of B-cell malignancies

We studied DNA methylation from two prospective cohorts where blood was collected from apparently healthy adult volunteers who were followed for up to 16 years. From these, 234 were selected who subsequently developed NHL, along with 236 who did not (Fig. 1, see Methods section for details), resulting in a total of 470 samples (Table 1). Genome-wide analysis of CpG methylation was performed on these samples using the Illumina HumanMethylation450k Beadchip, which assays >450,000 CpG sites. First, we sought to identify common biological themes related to future B-cell malignancy development using univariate linear regression applied to the whole cohort (470 subjects), assessing the relationship between each measured CpG site and future status (healthy vs NHL). DNA methylation estimates were obtained starting from the raw array data (Methods). After filtering out CpGs from sex chromosomes and CpGs in SNPs (single nucleotide polymorphisms), we identified 10,973 CpG sites whose methylation state was significantly associated with future development of B-cell malignancies (<0.05% False Discovery Rate, Supplementary Fig. 1, Supplementary Table 1a) after adjusting for age and sex. Among these, 4615 were hyper-methylated in subjects who subsequently developed NHL, and 6358 were hypo-methylated. CpG sites that were hyper-methylated in blood from subjects who developed future B-cell malignancies included many homeobox domain containing genes of the HOX family, as well as targets of the Polycomb repressive complex, mirroring differences previously described in lymphoma vs normal B cells^14^ (Supplementary Table 1b). 41 out of 45 significant CpGs in PAX gene loci were hyper-methylated in future lymphoma samples, including *PAX5*, a critical master regulator of B lymphocyte identity development and maintenance^15^. *TP53AIP1* and *TP53BP1* (hypo-methylated, in open sea regions) and *NFATC1* (hypo-methylated, in island regions), *MCL1* (apoptosis regulator and member of the *BCL2* family, hypo-methylated, in shore regions) and the tumor suppressor *BCL11B*, (hyper-methylated, in island regions) were amongst the genes which contain this predictive set of CpG loci (Supplementary Table 1a). These results support that there are methylation changes present in the blood in pathways related to B-cell malignancy development, that are detectable prior to overt disease presentation.

**Table 1:** Demographics of the study population (470 samples), comprising future Non-Hodgkin Lymphoma (NHL) and controls from two cohorts (Northern Sweden Health and Disease Study [NSHDS] and EPIC-Italy from the European Union 7th Framework Programme).

	Variable	Total population	NSHDS	EPIC
Sample size	Sample size	470	314	156
Status disease	Controls	236	159	77
	Future NHL	234	155	79
Sample size filtered*	Sample size	278	314	156
Status disease filtered*	Controls	139	96	43
	Future NHL	139	90	49
Subtypes NHL	B	41	32	9
	BCLL*	28	19	9
	DLBCL	40	29	11
	FL	33	14	19
	LPL	7	7	0
	MM*	67	46	21
	MCL	10	0	10
	LYM	8	8	0
Gender	Male	241	175	66
	Female	229	139	90
Age	Age;mean(SD)	52.48 (8.15)	51.20 (7.84)	55.05 (8.16)
BMI	BMI;mean(SD)	26.24 (3.88)	26.12 (4.09)	26.49 (3.42)

Characteristics of the population. B-cell chronic lymphocytic leukaemia (BCLL), diffuse large B-cell lymphoma (DLBCL), follicular lymphoma (FL), (lymphoplasmacytic lymphoma) LPL, multiple myeloma (MM), mantle cell lymphoma (MCL) and different B-cell lymphomas (BO, BALL, BNOS) and others (LYM). *BCLL and MM are distinct clinical entities from the remaining NHL types so these 95 subject and 95 controls were excluded to build predictive models, resulting in a total of 278 samples.

### Deconvolution identifies cell-specific pathway deregulation prior to NHL development

We estimated proportions of immune cells in blood samples using CIBERSORTx^16,17^ with a DNA methylation based signature matrix (Supplementary Table 1c) representing the cell types from ref. ^18^: neutrophils, eosinophils, basophils, monocytes, naïve B cells, memory B cells, T helper lymphocytes naive, memory helper T cells, regulatory T cells, T CD8 cytotoxic lymphocytes naive, cytotoxic CD8 memory T, and natural killer (NK) cells. CIBERSORTx is a computational framework that infers, in addition to cell fractions, cell-type-specific transcriptomes from RNA profiles of intact tissues. Here we extended the framework to estimate DNA methylation profiles of individual cell types within bulk profiles. Supplementary Table 1d summarizes immune cell fraction estimates from methylation-based deconvolution using CIBERSORTx. Consistent with expectations for peripheral blood, neutrophils are the most abundant cells (~50%), followed by memory T helpers (~12%), monocytes (~8%), cytotoxic CD8 memory T cells (~8%), NK cells (~5%), naïve T helper cells (~4%), eosinophils (~3%), memory B cells (~3%), naïve cytotoxic CD8 T cells (~2%), and B cells naive (2%), while basophils are the least abundant cell types (close to 0%) (Supplementary Fig. 2). We compared our estimates with a different method EpiDISH^19^ with an independent signature (see methods section for details), resulting in high correlations between our estimates and those obtained by alternative methods except for eosinophils (Pearson correlations: B cells = 0.97, CD8 T cells = 0.83, CD4 T cells = 0.89, monocytes = 0.90, neutrophils = 0.98, NK = 0.84 and eosinophils 0.37). Deconvolution using the Salas et al.^18^, library resulted in more reliable eosinophil estimates than those obtained from other libraries as reported in the original manuscript. Blood samples from the future-NHL group showed few differences from the control group, except for a trend towards higher memory B cells (*P* = 0.07) and T regulatory cells (*P* = 0.03) (Supplementary Table 1e). CIBERSORTx can estimate representative methylation profiles for individual cell types within bulk samples (*High-resolution* mode). We used this capability to test if methylation patterns within cell types can identify biological mechanisms associated with increased risk of NHL. We identified significant pathways amongst differentially methylated genes (evaluated by Wilcoxon rank sum test) between future-NHL and controls identified by CIBERSORTx. Among others, pathways related to pluripotency of stem cells (*q* = 0.002), WNT signaling (*q* = 0.001) and G protein coupled receptor signaling (*q* = 0.01) in memory B cells and NOTCH signaling in neutrophils (*q* = 0.01). We identified lymphoma genes enriched in several cell types (“Disease” category marked in red in column L from Supplementary Table 1b) by mining disease-associated genes from the *ToppGene* database^20^, notably from B-cells (“Diffuse Large B-Cell Lymphoma”, *q* = 4.66 × 10^−9^) indicating that abnormal DNA methylation from cell-specific DNA methylation profiles occurs at genes implicated in lymphoma prior to diagnosis of disease.

### Epigenetic timer of cancer (“epiTOC”)

A DNA methylation-based model called “epiTOC” (Epigenetic Timer Of Cancer; see Methods), which reflects a mitotic clock-like process encoding the number of cell divisions, has been used to relate DNA methylation alterations arising during cell division to disease risk^21,22^. The 385 CpG sites that comprise the EpiTOC clock are unmethylated in many different fetal tissue types, and since their methylation tends to increase with age (but is not directly correlated with biological age, Supplementary Fig. 3a), the presumption is that the EpiTOC value is a proxy of accelerated cellular mitosis^23^. We found that the future NHL group had significantly higher epiTOC levels than the control group (Wilcoxon, *P* = 3.4 × 10^−4^) (Supplementary Fig. 3b). Differences in epiTOC estimates between future-NHL and controls suggest that alterations that could influence biological mechanisms are already happening at an early stage, and could at least partly be related to an accelerated mitotic clock in subjects who will later develop NHL.

### Development of a DNA methylation-based future NHL predictor

Quantitative DNA methylation measurement at the single-CpG-site level provides a comprehensive view of epigenetic changes but poses challenges for building predictive models due to high dimensionality (*p* ≫ *n*). For subsequent analyses, we removed B cell chronic lymphocytic leukemia (BCLL) and multiple myeloma (MM), as these are distinct clinical entities from the remaining NHL types, after which 278 samples remained (139 future NHL, and 139 age- and sex-matched controls; Table 1). We then developed a model to identify which subjects are more likely to develop NHL versus those who are likely to remain healthy. We selected an optimal subset of CpG sites that involves a trade-off between maximizing performance for predicting future disease status, and creating a parsimonious model that mitigates against overfitting. To achieve this, we performed a two-step feature selection procedure on 70% of the dataset selected at random from each class (future cancer vs future healthy), holding out the remaining 30% of samples as test data. First, we identified 10,587 CpGs that were univariately associated with future NHL in the training set using a Gaussian model (<5% False Discovery Rate). Second, we applied penalized multivariate linear regression (Elastic Net, with alpha = 1) to the same training data to condense the 10,587 CpGs to a subset of 179 that are associated with future disease status. However, a Random Forest classifier built using these 179 CpGs in the training set did not accurately classify future NHL from controls in the test set (0.57 accuracy, Supplementary Table 1f). Models built on variables other than DNA methylation such as age, gender, BMI and immune cell fractions also yielded performance that was close to random (~0.50 accuracy in the test set).

**Fig. 2:**
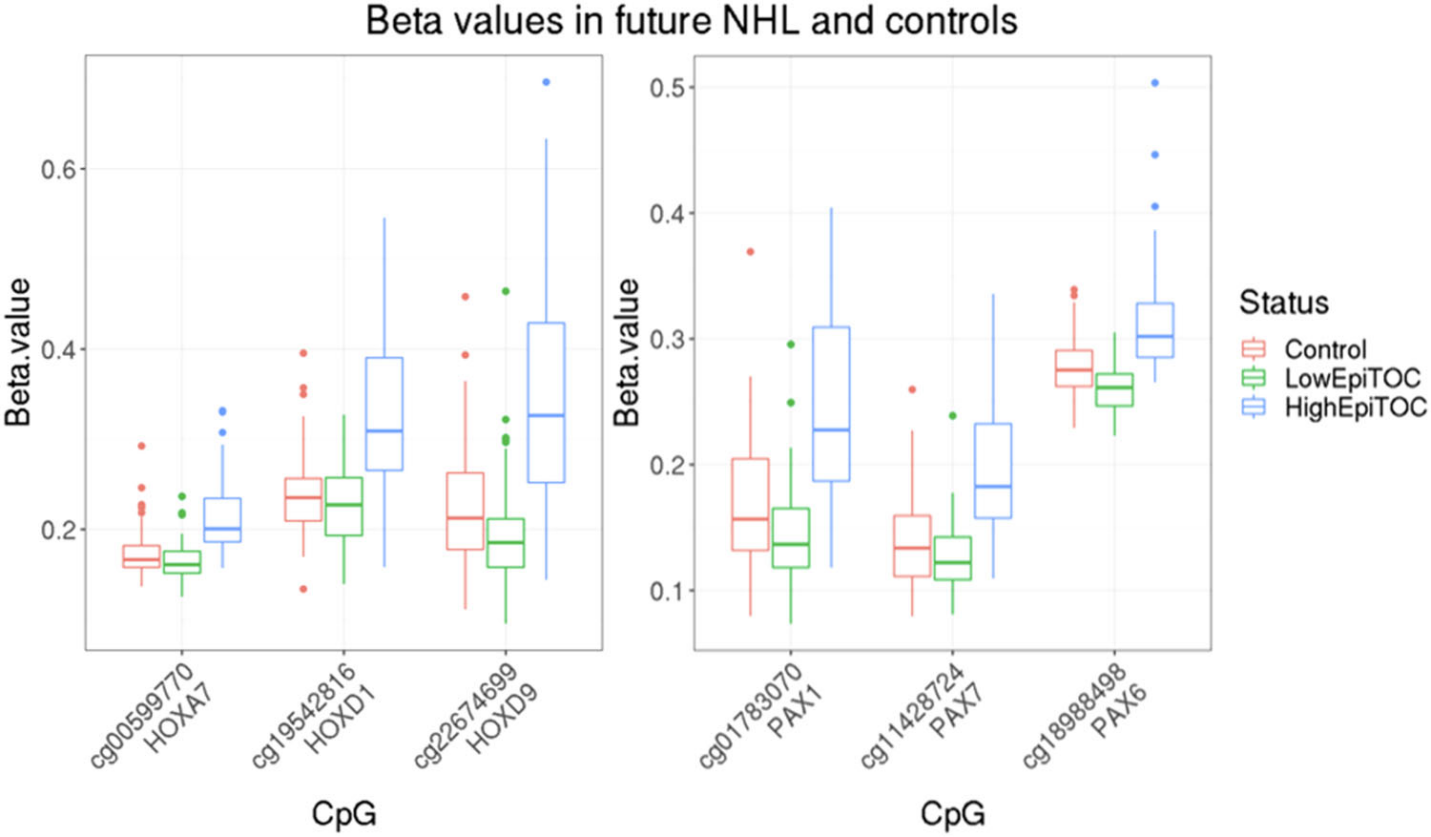
Top three CpGs corresponding to HOX and PAX genes in the prospective NHL and control groups. List of CpGs corresponding to HOX and PAX genes significantly hyper-methylated in the group with high epiTOC estimated levels (cg00599770 *P* = 1.34 × 10^−9^, cg19542816 *P* = 4.10 × 10^−8^, cg22674699 *P* = 1.22 × 10^−7^, cg01783070 *P* = 2.43 × 10^−8^, cg11428724 *P* = 3.75 × 10^−8^, cg18988498 *P* = 3.32 × 10^−8^) identified using univariate regression analyses (*N* = 278). The box plot uses the median (horizontal line), the first and third quartiles (ends of box) and points more than 3/2 times the interquartile range (dots).

When examining which subjects were misclassified, we noted that there were differences in epiTOC estimates between misclassified subjects and correctly classified subjects (*P* = 5.7 × 10^−4^ before adjusting for age and *P* = 5.0 × 10^−4^ after adjusting for age). We did not observe significant differences between misclassified and correctly classified subjects for other variables such as age, sex, memory helper T cells, cytotoxic CD8 memory T cells, and naïve B cells. Interestingly, the observed differences between misclassified subjects in epiTOC estimates (*P* = 5.7 × 10^−4^) revealed that true positives had higher epiTOC values than false negatives. We also observed that subjects with high epiTOC showed more significant hyper-methylation in CpGs from genes known to be important regulators of B lymphocyte development than the CpGs from subjects with low epiTOC values (Fig. 2). These included *HOXA7, HOXD9, HOXD1, HOXA9, HOXD8, HOXA10, PAX1, PAX6, PAX7, PAX3, PAX5,* and *PAX9*.

### Accounting for mitotic clock improves prediction of future NHL

We hypothesized that early deregulation of epiTOC indicating deviations in DNA methylation in mitotically-aged B-cells might precede proliferation and unrestricted division in future NHL, even before diagnosis. We further reasoned that assigning future NHL cases to different groups based on epiTOC might improve classifier performance as compared to the approach where we assume all future cases can be represented as one group with similar risk of developing lymphoma. Thus, we subdivided the future cases NHL into high epiTOC (epiTOC above its median value of 0.18) and low epiTOC (epiTOC < = 0.18) within the training set (Fig. 1). We next identified 1,572 CpGs that were univariately associated with the three-level categorical variable future NHL/high-epiTOC, future NHL/low-epiTOC, or control, using limma. We used a strict permutation-derived FDR cutoff^24^ appropriate for DNA methylation, of 2.4 × 10^−7^. We tested ten additional random cross-validation splits to check the stability of the findings by comparing the z-scores for each of the 10 random splits with the split that we used to obtain our predictive model, resulting in highly correlated z-scores for each CpG site and large overlap in the list of significant CpG sites (Supplementary Fig. 4). We then applied penalized multivariate linear regularized regression (Elastic Net, with alpha = 0.5) on the same population to obtain a more parsimonious set of 135 CpGs.

**Fig. 3:**
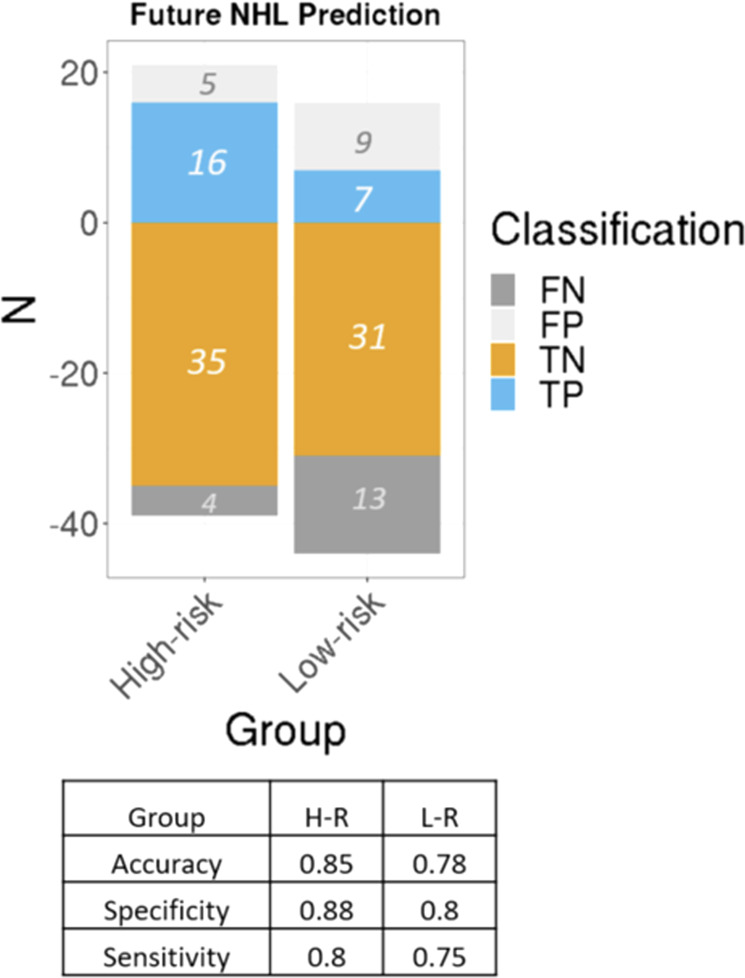
Performance of the predictive models using the bulk DNA methylation. Numbers are expressed in percentage and refer to the samples from the test set of the prospective cohort.

We applied random forests to derive a final classification model from the training set using the three-level outcome future NHL/high-epiTOC, future NHL/low-epiTOC, or control as outcome and tested it on the left-out 30% of samples. We refer to the resulting per-subject score as the Future LYmphoma Predictor or FLYP. The performance of the predictive model was 85% accuracy for the high epiTOC group and 78% for the low epiTOC group in the test set, with high sensitivity and specificity (Fig. 3). Different epiTOC cutoff values other than the median resulted in similar performances around the median value and worse performance for more distant values (Supplementary Table 1g). Our classifier showed high specificity and sensitivity (88% and 80%, respectively) for future NHL subjects with high epiTOC value, as well as future-NHL with low epiTOC (80% and 75%, respectively) (Fig. 3).

Most of the loci included in FLYP are found in CpG islands near the transcription start sites of genes, especially those with higher predictive importance (Supplementary Table 3a). The most predictive CpG is hypo-methylated in the future-NHL/high-epiTOC group and located in CpGs islands and targets the gene *VGLL3*, which is known to be involved in tumor cell proliferation^25^ associated with future-NHL/high-epiTOC NHL group. Several transcription factors from the TEAD family which are downstream effectors of the Hippo pathway, and for which *VGLL3* is a cofactor, are also significantly different between future NHL and controls (hyper-methylated in the future-NHL/high-epiTOC).

### Changes in blood immune composition are associated with future NHL development

Models based on cell fractions, age and sex as features instead of CpGs yielded NHL predictions better than random (70% and 61% accuracy for predicting high and low epiTOC future NHL, respectively) but less accurate than the CpG-based model. The future-NHL/high-epiTOC score and the EpiTOC values were positively correlated with inferred fractions (by deconvolution) of cytotoxic memory CD8 T cell, memory B cell, regulatory T cell, and NK cell fractions; and negatively correlated with neutrophil, naïve T helper cell, naïve cytotoxic CD8 T cells and naïve B cell fractions (Supplementary Fig. 5). The future-NHL/low-epiTOC group has a negative correlation with cytotoxic memory CD8 T cells and positive correlation with neutrophils. Moreover, the future-NHL/low-epiTOC group has statistically significantly higher neutrophil to lymphocyte ratios than the control group (*P* = *0.006*). The higher memory B cell fractions in future-NHL samples suggests possible higher proliferation of B cells prior to NHL development. In addition, higher levels of NK cells in peripheral blood have been observed in newly diagnosed DLBCL patients as compared to controls^26^. Together, these analyses identify differences in blood immune composition associated with elevated NHL risk. However, use of artificial intelligence approaches applied to DNA methylation improve predictions above those associated with immune composition, age and sex, supporting the utility of FLYP.

### FLYP foreshadows DNA methylation changes present in NHL

We hypothesized that the FLYP could be capturing low levels of epigenetic deregulation that occur during early NHL tumorigenesis, which become more pronounced in overt disease. We therefore computed FLYP for samples in five cohorts of tumor biopsies or blood samples from adults who currently had NHL. We also interrogated a large cohort of healthy subjects (healthy at the moment of blood sampling, but lacking follow-up information) to evaluate the predicted proportion of subjects at risk of developing NHL in the entire population (details in Supplementary Table 3b). On average 96.3% of the samples from the NHL cohorts and only 4.4% from the current healthy cohort were classified as future-NHL/high-epiTOC (Supplementary Table 3c).

EpiTOC estimates from all current-NHL cohorts were significantly higher than those from the future-NHL cohort or current healthy controls (Supplementary Fig. 6); likewise epiTOC estimates from the NHL prospective group were significantly higher than those from the prospective controls (Wilcoxon, *P* = 4.4 × 10^−5^). However, EpiTOC estimates alone did not yield accurate NHL predictions (56% accuracy).

### Shared methylation changes across disease subtypes prior to NHL development

NHL comprises a heterogeneous group of neoplasms of the lymphoid system, therefore we tested the ability of methylation to classify future NHL across subtypes defined according to SEER ICD-0-3 morphology codes^27^. We conducted differential methylation analysis on future NHL vs control for each subtype (Supplementary Fig. 7). The z-scores assessing significance of differential methylation between future NHL and controls at each CpG site obtained from each individual subtype correlated differently across subtypes. The most pairwise correlated z-scores corresponded to the subtypes ML and LYM (Pearson correlation = 0.5), while the least correlated z-scores corresponded to LYM and DLBCL or LPL and DLBCL (Pearson correlation = 0.08). To further test if some NHL subtypes have more generalizable predictive power than others, we trained models for each NHL subtype on the other subtypes and then tested them on the “test” one (leave-one-subtype-out). Future development of LPL (lymphoplasmacytic lymphoma, *N* = 7, accuracy future-NHL/high-epiTOC group = 0.8, accuracy future-NHL/low-epiTOC group = 0.94), DLBCL (diffuse large B-cell lymphoma, *N* = 40, accuracy future-NHL/high-epiTOC group = 0.79, accuracy future-NHL/low-epiTOC group 0.74), LYM (others, *N* = 8, accuracy future-NHL/high-epiTOC group = 0.69, accuracy future NHL/low-epiTOC group = 0.81) and FL (follicular lymphoma, *N* = 7, accuracy future-NHL/high-epiTOC group = 0.78, accuracy future-NHL/low-epiTOC group = 0.75) subtypes showed better performance from models built on other subtypes as training sets than MCL (mantle cell lymphoma, *N* = 10) (Supplementary Table 3d).

Most B‐cell neoplasms originate from the germinal center reaction as a result of mutation of genes involved in epigenetic regulation and immune receptor signaling^28^. We aligned the epiTOC values from all subtypes (including B-cell chronic lymphocytic leukaemia [BCLL] and multiple myeloma [MM]) with the development of the B-cell repertoire in the germinal center and the attributed origin of each NHL subtype (Supplementary Fig. 3b). MCL showed on average the lowest epiTOC values, while BCLL (the most frequent type of B-cell leukemia), LPL and DLBCL showed the largest epiTOC values. Naïve B cells aggregate outside of the germinal center leading to the formation of the mantle zone where MCL develops, while BCLL and DLBCL occur at the stage of differentiation prior to plasma cell and memory B-cell development^28^.

### Overrepresentation and hyper-methylation of polycomb-target promoter CpG sites in the future-NHL DNA methylation signature

**Fig. 4:**
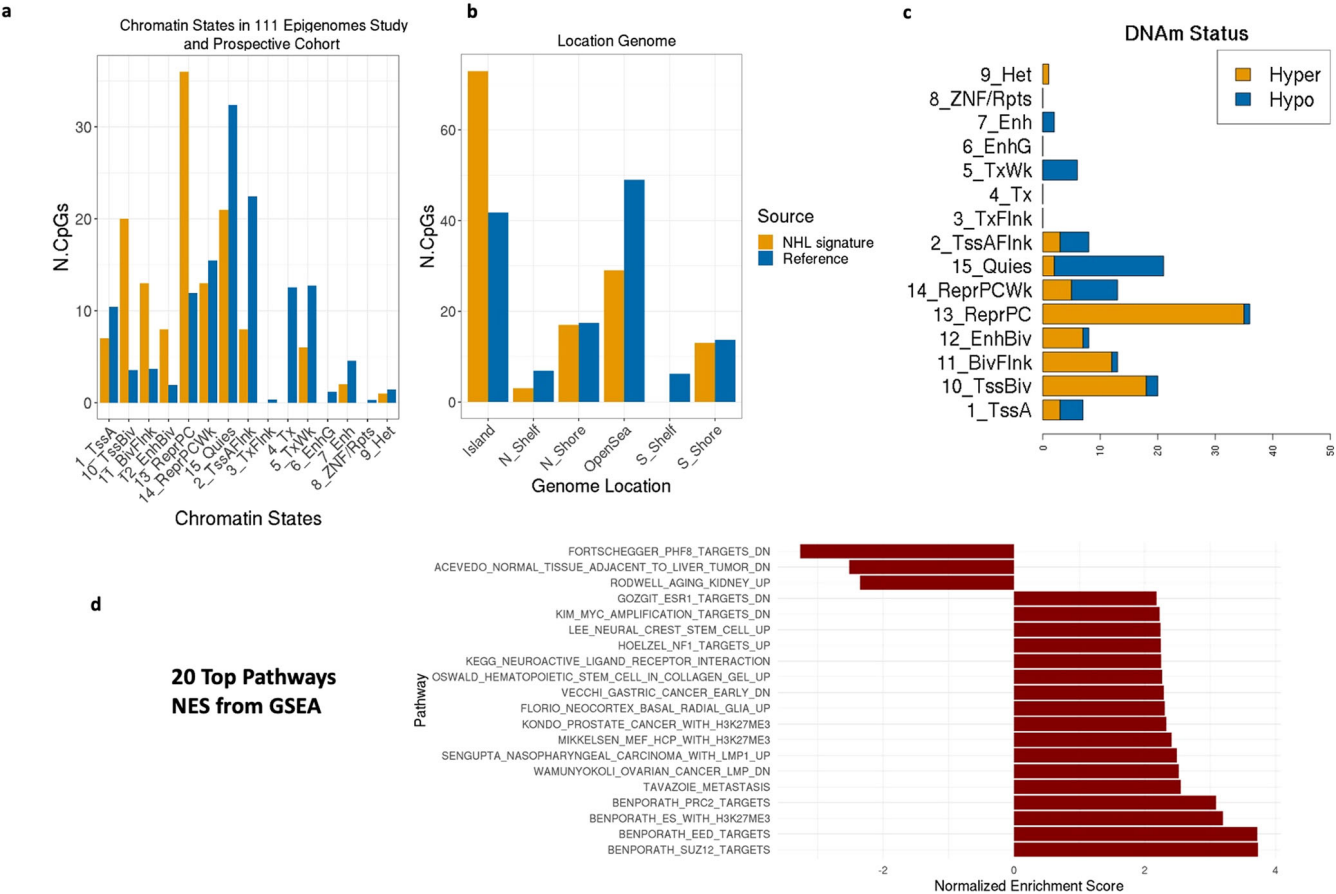
Landscape of the future NHL signature. **a** Number of CpGs from the signature from each chromatin state compared to the annotated genome. **b** Location of the CpGs from the signature compared to the annotated genome. **c** Number of hyper-/hypo-methylated CpGs from the signature from each chromatin state within the prospective cohort (reference: healthy controls from the prospective study). **d** Top 20 pathways from GSEA analysis (C2-curated gene sets).

To investigate the potential functional significance of the 135 CpGs comprising FLYP (Supplementary Table 3a), we compared the CpGs in it with ones associated with 15 specific chromatin states defined by the 111 Epigenomes study, derived from primary mononuclear cells (E062) using 450 k arrays^29^. The number of CpGs from the signature that belong to regions associated with de-repression (‘TssBiv’, FDR: 9.31 × 10^−19^) and repressed by polycomb proteins or the “ReprPC” chromatin state (FDR: 1.64 × 10^−11^) are larger than expected by chance (significance levels calculated by comparing the distribution of chromatin states in the FLYP signature with 1000 lists of randomly selected CpGs) (Fig. 4a and Supplementary Table 3e). Most of the CpGs from the signature are located in CpG islands (*P* = 6.03 × 10^−9^) (Fig. 4b and Supplementary Table 3e). Polycomb-mediated repression of expression is generally associated with hypermethylation of target genes, as observed in the top 50 differentially methylated CpGs from the signature (Fig. 4c, Supplementary Fig. 8a). The signature also shows enrichment of the TssBiv chromatin state, which has been associated with embryonic stem cells and induced pluripotent stem cells in other studies^30^. In order of significance, the top 5 pathways from GSEA analysis are: BENPORATH_SUZ12_TARGETS (targets of the Polycomb protein SUZ12 in human embryonic stem cells), BENPORATH_EED_TARGETS (targets of the Polycomb protein EED in human embryonic stem cells), BENPORATH_ES_WITH_H3K27ME3 (genes possessing the trimethylated H3K27 mark in their promoters in human embryonic stem cells), BENPORATH_PRC2_TARGETS (Polycomb Repression Complex 2 (PRC) targets identified in human embryonic stem cells, and TAVAZOIE_METASTASIS (genes up-regulated in metastatic cell lines of lung and bone relative to the parental line of breast adenocarcinoma) (Fig. 4d). Overexpression of genes normally enriched in embryonic stem cells and repression of Polycomb-regulated genes has been associated with histologically poorly differentiated tumors across different cancer types, and poorly differentiated tumor cells in NHL are associated with fast growing and aggressive tumors^31^. Genes targeted by CpGs significantly associated with future-NHL are also enriched in pathways related to T cell activation (Supplementary Fig. 8b).

## Discussion

Low levels of molecular signals, small effect sizes, population heterogeneity, and relatively small groups of subjects from long-term prospective studies are major challenges inherent in cancer prediction. Furthermore, some subjects might not have developed NHL-related molecular alterations at time of sample acquisition, hampering the classification task. We developed a peripheral blood-based DNA methylation signature (FLYP) based on 135 key CpG sites that can predict NHL development, with an accuracy of 85% for the future-NHL/high-epiTOC group and 78% for the future-NHL/low-epiTOC group. We built a comprehensive computational framework to condense the original DNA methylation data into a lower-dimensional feature space to develop the prospective signature, by studying differences in DNA methylation levels from blood samples between a future NHL group (lead time of 16 years) and healthy control group. Notably, FLYP performance was improved by stratifying the population into high-epiTOC and low-epiTOC individuals, reflecting accelerated mitosis of cells. Clearly this require validation in an external prospective cohort but nevertheless this observation was crucial to identifying a predictive model. We performed several tests to confirm that FLYP predicts mostly “control” or low-epiTOC in an independent population of 656 healthy subjects; and that it predicts patients who currently have NHL in tissue samples from four independent cohorts.

While immune cell fractions, age and gender have some degree of predictive power, the combination of epiTOC and FLYP outperforms the other models or a model based on epiTOC alone. An elastic net baseline model resulted in worse performance (81% accuracy for high epiTOC group and 68% accuracy for low epiTOC group) than Random Forests. The association between NHL risk and epiTOC is consistent with other studies reporting accelerated mitosis based on methylation in cancer tissue samples, cancer cell lines, pre-cancerous lesions and normal epithelial cells exposed to a major carcinogen^31^. It has been suggested that accelerated cell division could lead to epigenetic cellular heterogeneity and predispose the tissue to future neoplastic transformation^21,32–34^. The proliferation of these new clones could be responsible for the increased cellular proliferation and epigenetic changes observed in active NHL subjects. DNA methylation profiles may implicitly capture some of the complexity of cell heterogeneity (both cell abundance and molecular profiles) that is not captured when using cell type fraction estimates, facilitating good model performance.

Overrepresentation of the “ReprPC” chromatin state with hyper-methylation CpG sites in our DNA methylation signature indicates polycomb-mediated repression, which usually targets pro-differentiation genes in stem cells and cancer. Hyper-methylation potentially silences differentiation-related genes in effector lymphocytes, inducing a more undifferentiated, stem-like cell state. This hyper-methylation might be mechanistically relevant for NHL initiation, and lead to higher epiTOC values observed in active NHL/future NHL subjects. We also found B-cell lymphoma related key genes such as *HOX* and *PAX5* deregulated at the DNA methylation level in future NHL compared to controls. Overall, our study suggests a biologically informative DNA methylation signature that reflects B-cell deregulation before NHL diagnosis. This also supports the idea that the predictive features represent biological mechanisms leading to increased risk of NHL, rather than being passive markers of neoplasticity.

FLYP has the potential to be a convenient and clinically applicable risk score for predicting NHL using DNA methylation. Future prospective studies will be required to confirm its utility and incorporate these results into clinical practice, for example to recommend that people with high risk have more regular checkups. Since only <2% of CpG sites are covered by the Illumina HumanMethylation450k Beadchip, the predictive value of FLYP would likely be improved by generating new datasets using a sequencing-based methylation method. In addition to a predictive NHL signature, we provide our computational framework as a resource to identify top performing approaches across pre-processing, normalization, feature selection, data transformation, predictive models, deconvolution analyses and omics integration. This should also facilitate reproducible DNA methylation analyses using new data sets.

In conclusion, we built a score based on DNA methylation from peripheral blood samples that can predict future (up to 16 years) development of NHL. This study demonstrates the potential for early detection to enable early treatment of NHL that may improve outcomes of patients.

## Methods

### Study cohorts

The study population is based on participants from two existing prospective cohorts: the Italian component of the European Prospective Investigation into Cancer and Nutrition (EPIC‐Italy)^27^ and the Northern Sweden Health and Disease Study (NSHDS)^35^. After providing written informed consent, blood samples were prospectively collected from subjects who were healthy at enrolment (47,749 volunteers within the EPIC-Italy study and 80,000 subjects within the NSHDS study). Instances of NHL occurring during the study period (16 years) were identified through local Cancer Registries (loss to follow-up < 2%). This study was approved by the committee on research ethics at the relevant institutions in accordance with the Declaration of Helsinki of the World Medical Association. All participants provided written informed consent to take part in the study at recruitment.

For each NHL case identified within the two cohorts during follow-up, one random control was selected among all cohort subjects free of cancer at the time of diagnosis, matched by cohort, center, gender, date of blood collection (±6 months) and age at recruitment (±2.5 years). Participants diagnosed with disease within less than two years of blood sample collection were excluded.

### Sample preparation

Buffy coats were isolated from the collected blood samples within 2 h of blood collection for both cohorts, and placed in long-term cold storage (liquid N_2_ in EPIC- Italy and −80 °C in NSHDS). Careful evaluation of the data from a pilot study experimenting with the conditions of sample collection did not reveal any systematic influence of storage time on epigenomic profiles^36^.

The final number of included successfully analyzed samples was 234 NHL cases and 236 matched controls. The characteristics of the study population are summarized in Table 1.

### Preprocessing DNA methylation

Genome-wide analysis of CpG methylation was conducted on the Illumina HumanMethylation450k Beadchip. We used the “preprocessQuantile” function from the “minfi 1.32.0” R package to normalize the data. We corrected M values for batch effects (adjusting for the variable corresponding to the date of chip analysis) using the function “ComBat”^37^ from the “sva 3.34.0” R package. Methylation levels were expressed as beta values (ranging from 0 to 1, corresponding to the proportion of methylation at each of the >450,000 genomic CpG sites measured by the Infinium platform) for visualization and epigenetic clock calculations and M-values (corresponding to the logarithmic ratio of the methylated versus the unmethylated signal intensities at each site) for construction of predictive models. Because SNPs near the CpG site may alter methylation levels, we removed CpG at SNPs with a minor allele frequency >1% from the dbSnp database^38^. We also filtered out CpGs from sex chromosomes.

### Estimation of cell-type composition and cell type specific DNA methylation profiles

We used the deconvolution algorithm CIBERSORTx^16,17^ in combination with a previously defined signature matrix based on epigenetic profiles to infer the proportions of cell-types present in the blood samples, and estimate the DNA methylation profiles for each immune cell-type. The cell composition was estimated using methylCIBERSORT (“FeatureSelect.V4” function) and a signature obtained from sorted cells from ref. ^18^, (GSE167998). We used the *High-Resolution* mode of CIBERSORTx to impute sample-level DNA methylation profiles using CpGs located in CpG islands and Wilcoxon rank sum test to identify differentially methylated CpGs in each imputed cell-specific DNA methylation profiles. Then, we used *ToppGene*^20^ and *GoMeth*^39^ for pathway analysis.

The immune cell fractions inferred from deconvolution were compared with those generated from a different algorithm called EpiDISH based on Robust Partial Correlations^19^ and different data: purified normal blood cell sub-populations white blood DNA methylation data derived from granulocytes (12), CD8 + (cytotoxic T-lymphocytes) (6), CD4 + (cytotoxic T-lymphocytes), CD19 + (B-lymphocytes) (6), CD56 + (NK cells) (6), CD14 + (monocyte lineage) (6) and eosinophils^40^ as a sanity check of the first of the deconvolution involving estimation of cell fractions.

### Epigenetic age and DNAm-based age-correlative model “epiTOC” (Epigenetic Timer Of Cancer)

The number of cell divisions per stem cell is an important cancer hallmark. We computed estimates of the rate of stem cell division using a DNA methylation-based age-correlative model based on the EpiTOC signature^21^. EpiTOC correlates with chronological age to a lesser extent than other epigenetic clocks that aim to predict age (Supplementary Fig. 3a). We tested if epiTOC is higher in active NHL patients compared to future NHL or healthy subjects using the Wilcoxon signed-rank test.

### Differential DNA methylation to identify biological themes associated with future NHL development

We identified CpG sites significantly associated with future development of NHL (<5% False Discovery Rate, Supplementary Table 2a), as described further below. We used *ToppGene*^20^ and *GoMeth*^39^ for pathway analysis and Gene Set Enrichment Analysis (GSEA)^41^ to further investigate whether pre-defined sets of genes from the MSigDB database (C2-curated gene sets and C7-immunologic gene sets) show statistically significant and concordant differences between the future NHL and control subjects.

### Two-step feature selection

We first split the data into 70% training and 30% test, balanced for the three classes (future NHL with high EpiTOC, future NHL with low EpiTOC and control status) distributions within the split, and then used linear regression models using limma from the ‘missMethyl_1.20.4’ R package^42^ to investigate the relationship between each individual CpG DNA methylation probe from the training set and the NHL outcome (control group as reference). We fit models using least squares regression on all CpGs separately, and accounted for multiple hypothesis testing using a 2.4 × 10^−7^ cutoff specific for DNA methylation based on permutations^24^.

Then, we selected the common CpGs significantly associated with both high and low EpiTOC future NHL groups and performed regularization using elastic net (which combines the L1 and L2 penalties of the lasso and ridge methods) methods^43,44^ with the same split as the univariate approach to select a subset of the CpGs. We efficiently maximized the amount of joint information relative to the status of future disease by identifying the top common features with non-zero coefficients.

### Constructing an AI-based prospective diagnostic system

We built random forest classification model using the same training data from the split in the initial two-step feature selection. We assessed performance of the final model fit on the left-out test data. We used “Random Forest“ model from the “randomForest 4.6–14” R package, implemented in the R package “caret 6.0–86”. To train the models, three separate 10-fold cross-validations were used as the resampling scheme. We set to 30 the parameter that defines the total number of parameter combinations that will be evaluated (“tuneLength”). We tuned hyper-parameters to assess the model fit on subsets of the training dataset and we reported the results from the test set to assess the performance on an independent set.

We assessed the performance of the classifier using the following metrics: accuracy (percent of correct classifications obtained), sensitivity (ability to detect future disease in the population of future diseased individuals), specificity (ability to correctly rule out the future disease in a disease-free population), F1 Score = 2 * Precision * Recall/(Precision + Recall) and AUC value of the resulting ROC curves.

We also tested if the combination of gender, age, epigenetic age, epiTOC and proportion of cell-types or some of these variables alone had predictive power by training models using these variables as features, with future case/control status as the dependent variable.

### Confirmation of the signature on NHL subjects and healthy subjects

We evaluated our predictive model in four cohorts of subjects with current NHL, and in a cohort of 656 healthy subjects (at the time of blood sampling – no follow up is available), utilizing genome-wide DNA methylation data from the same platform as our prospective cohort were available (Supplementary Table 3b). These validation datasets are different from the test dataset of the prospective cohort, which is also held back from the training of the model.

### NHL subtypes

NHL future cases were classified into subtypes according to the SEER ICD-0-3 morphology codes^27^. These include B-cell chronic lymphocytic leukaemia (BCLL), diffuse large B-cell lymphoma (DLBCL), follicular lymphoma (FL), (lymphoplasmacytic lymphoma) LPL, multiple myeloma (MM) and mantle cell lymphoma (MCL), heterogeneous category of B-cell malignancy and others (LYM) (Table 1). We built predictive models leaving-one-subtype-out, where the training set comprised all NHL subtypes but one and the test set the remaining subtype. BCLL and MM cases were removed from the final analyses to build predictive models as they are clinically distinct from the other disease entities.

### Description of the DNA methylation signature

We investigated whether methylation signatures were statistically enriched for a comprehensive set of previously defined DNA chromatin features based on methylation profiles. We used the 15 chromatin states from the 111 epigenomes study^29^ which were derived from primary mononuclear cells (E062) using 450k arrays.

We studied the location of the CpGs from the signatures and characterized the proportion of CpGs from islands, shores, shelves, and open seas.

We calculated significance levels by comparing the distribution of chromatin states and genome location of the CpGs from the FLYP signature with 1000 lists of the same number of CpGs randomly selected from the entire genome.

### Reporting summary

Further information on research design is available in the Nature Research Reporting Summary linked to this article.

## Supplementary information

Supplemental Material

Data Set 1

Data Set 2

Data Set 3

REPORTING SUMMARY

## Data Availability

The data is available at 10.5281/zenodo.6578747 and https://github.com/alespe/PredDNAm. A portion of the dataset is not publicly available due to restrictions imposed by Swedish legislation on the protection of personal data, but is available under request (contact person Ingvar Bergdahl, ingvar.bergdahl@umu.se). The validation datasets are GSE40279^45^, GSE37362^46^, GSE42372^47^, GSE109381^48^ and The Cancer Genome Atlas (TCGA) DLBC (https://www.cancer.gov/tcga).

## Code availability

The code implementation is publicly available at https://github.com/alespe/PredDNAm.
