# Supplementary material for: Peripheral blood DNA methylation profiles predict future development of B-cell Non-Hodgkin Lymphoma
Source: NPJ Precis Oncol. 2022 Jul 21;6:53. doi: 10.1038/s41698-022-00295-3 (PMC9304422; doi:10.1038/s41698-022-00295-3)

Supplementary Information

Supplementary Figure 1.DNA methylation heat maps normalized and mean centered from future NHL samples and controls of the significant CpGs sites (<1% False Discovery Rate) using Gaussian models, including mitotic clock (EpiTOC) range (see Methods section for details).

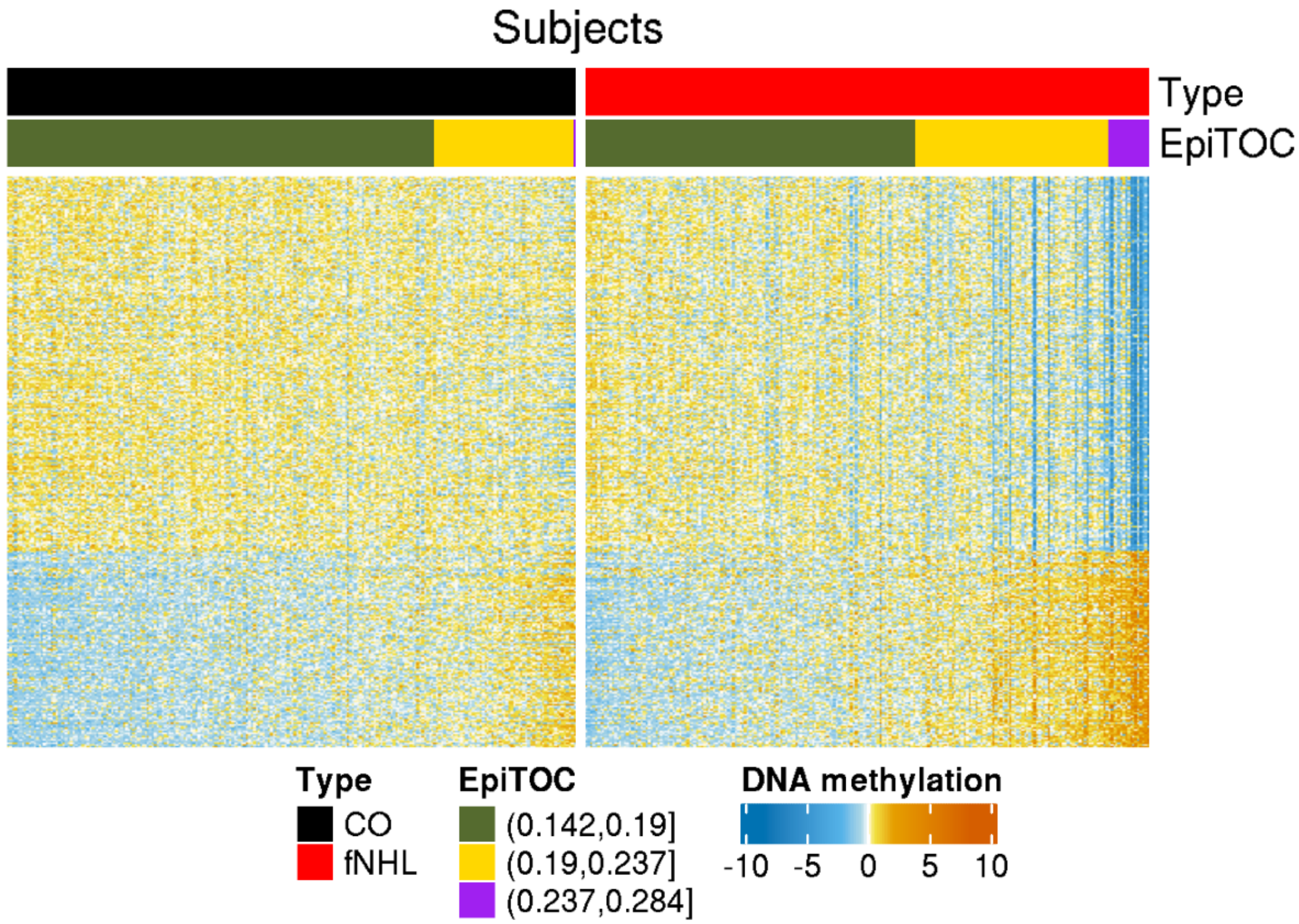

Supplementary Figure 2. Immune cell fractions across all subjects (left) and subgroups (right).

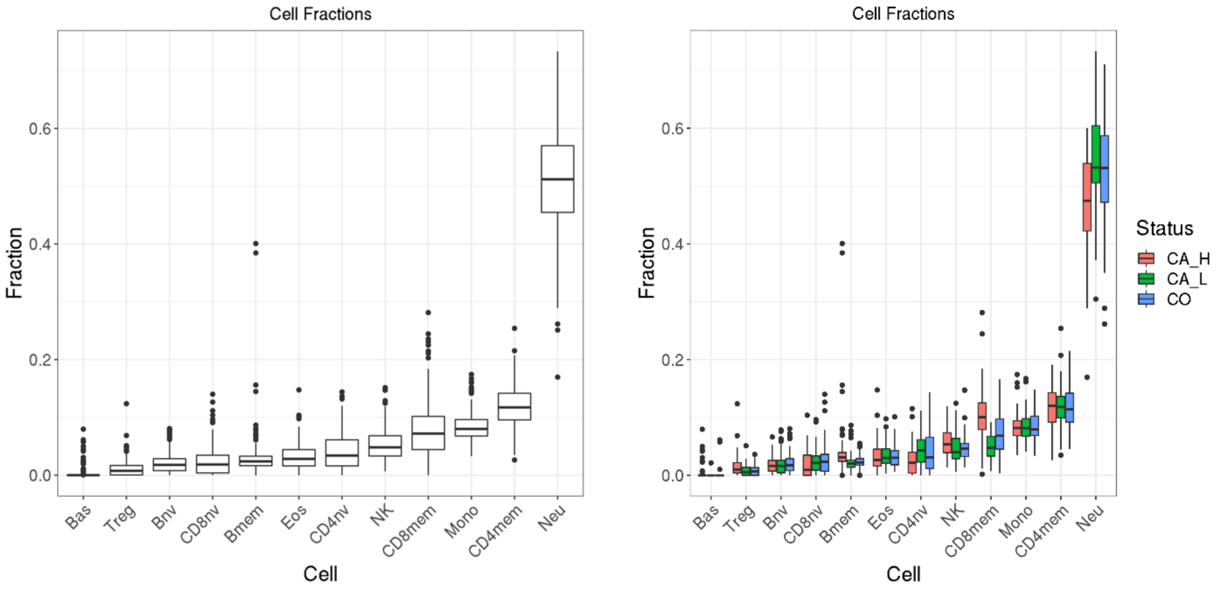

x-axis of the box plot represents the immune cell type y-axis the relative fraction from 0 to 1 estimated from bulk DNA methylation data using CIBERSORTx. The box plot uses the median (horizontal line), the first and third quartiles (ends of box) and points more than 3/2 times the interquartile range (dots).CA_H=Future NHL with high EpiTOC values. CA_L=Future NHL with low EpiTOC values. CO=controls. Neu=neutrophils, Eos=eosinophils, Bas=basophils, Mono=monocytes, Bnv=B lymphocytes naive, Bmem=B lymphocytes memory, CD4nv=T helper lymphocytes naive, CD4mem=T helper lymphocytes memory, Treg=T regulatory cells, CD8nv=T cytotoxic lymphocytes naive, CD8mem=T cytotoxic lymphocytes memory, NK= natural killer lymphocytes.

Supplementary Figure 3. Relationship between age and epigenomics clocks (A). EpiTOC levels in the prospective cohort across controls and future NHL (left) and controls and different future NHL subtypes (right, Wilcoxon, $P=3.4x10^{-4}$) (B). *The box plot uses the median (horizontal line), the first and third quartiles (ends of box) and points more than 3/2 times the interquartile range (dots).*

A)

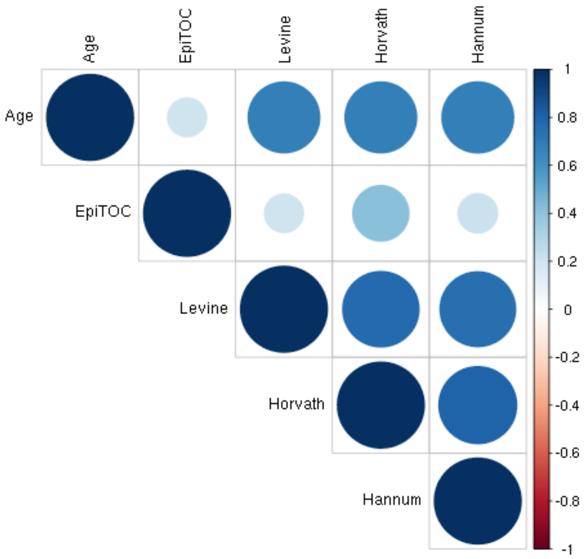

B)

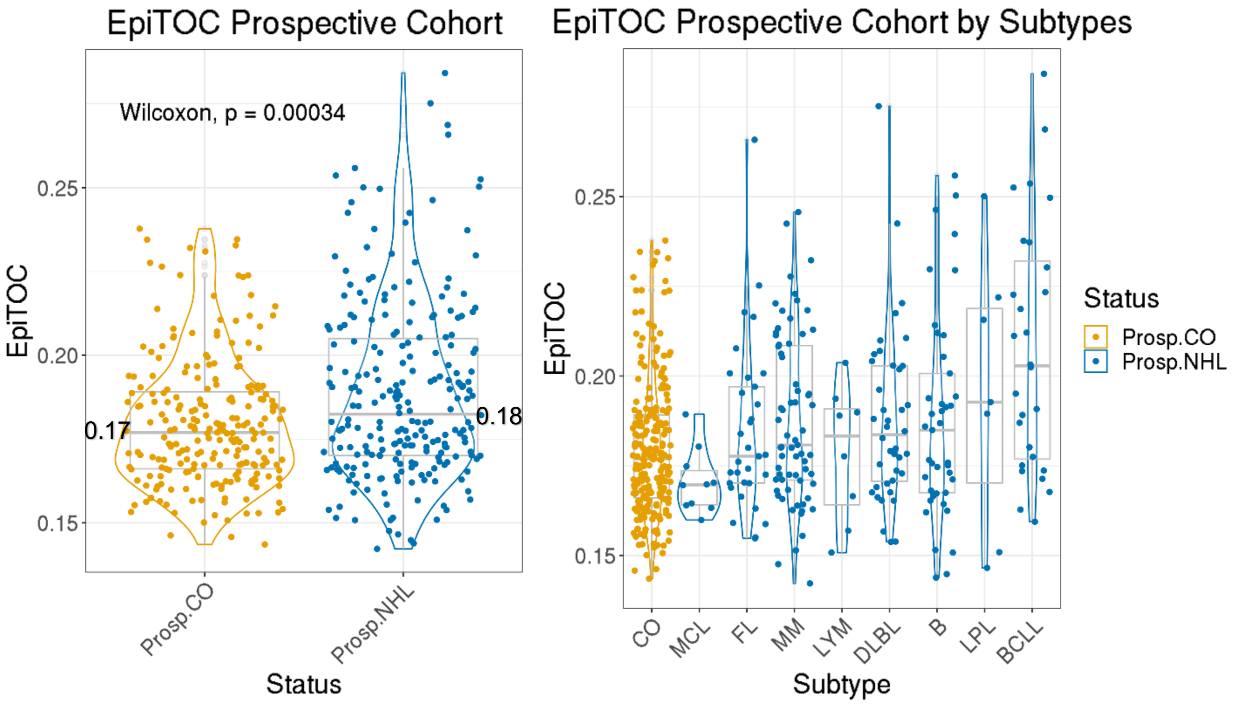

Supplementary Figure 4. 10-fold cross validation: Intersection of significant CpGs (<5% False Discovery Rate) across ten random splits of the prospective cohort dataset comprising 278 samples (A) and correlation of z-scores (B)

A)

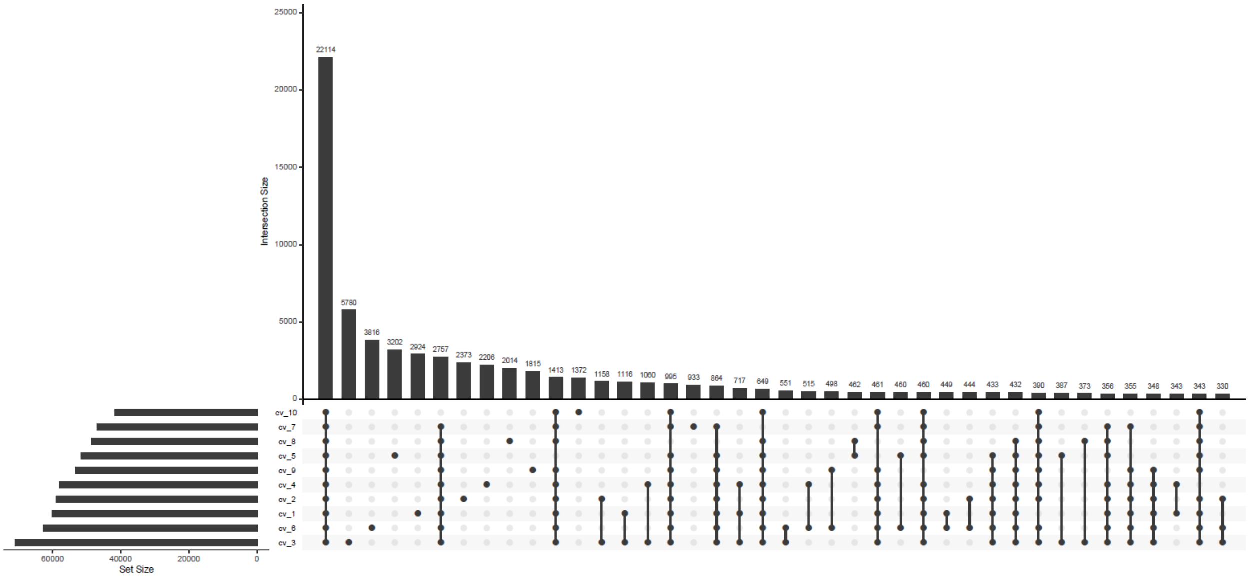

B)

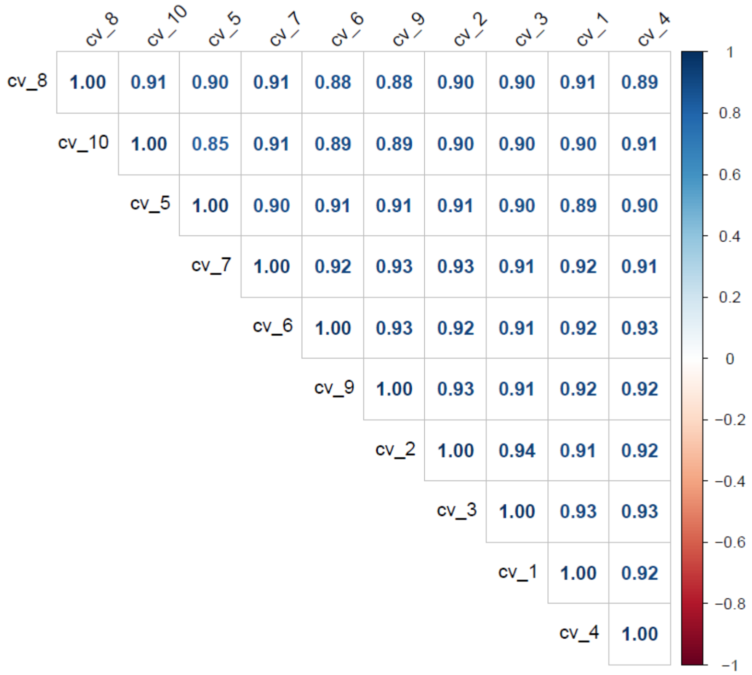

Supplementary Figure 5. Correlation across predictive scores (HR=predictive score for high epiTOC group, HL=predictive score for low epiTOC group) and estimated immune cell fractions.

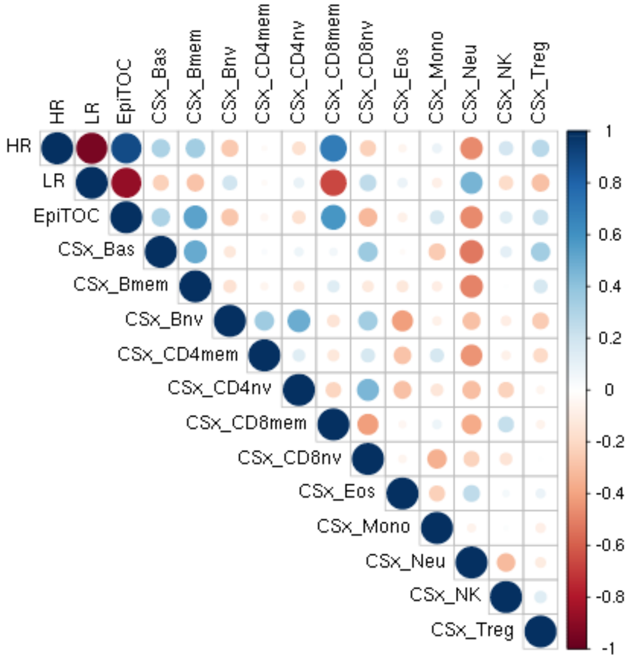

Neu=neutrophils, Eos=eosinophils, Bas=basophils, Mono=monocytes, Bnv=B lymphocytes naive, Bmem=B lymphocytes memory, CD4nv=T helper lymphocytes naive, CD4mem=T helper lymphocytes memory, Treg=T regulatory cells, CD8nv=CD8 T cytotoxic lymphocytes naive, CD8mem=CD8 T cytotoxic lymphocytes memory, NK= natural killer lymphocytes.

Supplementary Figure 6. EpiTOC estimates across the independent cohorts (GSE109381, GSE37362, TCGA_DLBC, GSE42372 and GSE40279, details in Table S3b) and the prospective cohort. Statistics based on Wilcoxon tests. Prosp.CO=Controls from the prospective cohort. Prosp.LY= Future NHL from the prospective cohort. *The box plot uses the median (horizontal line), the first and third quartiles (ends of box) and points more than 3/2 times the interquartile range (dots).*

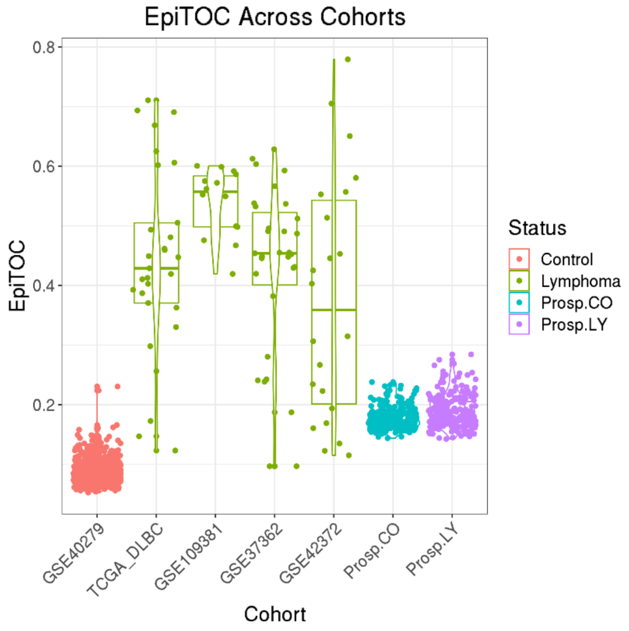

Supplementary Figure 7. Correlation z-scores from all CpGs by running regression analysis for each subtype (outcome future NHL/control, reference=control).A) Correlation plots. B) Correlation tables. B-cell chronic lymphocytic leukaemia (BCLL), diffuse large B-cell lymphoma (DLBCL), follicular lymphoma (FL), (lymphoplasmacytic lymphoma) LPL, multiple myeloma (MM), mantle cell lymphoma (MCL) and different B-cell lymphomas (BO, BALL, BNOS) and others (LYM).

A)

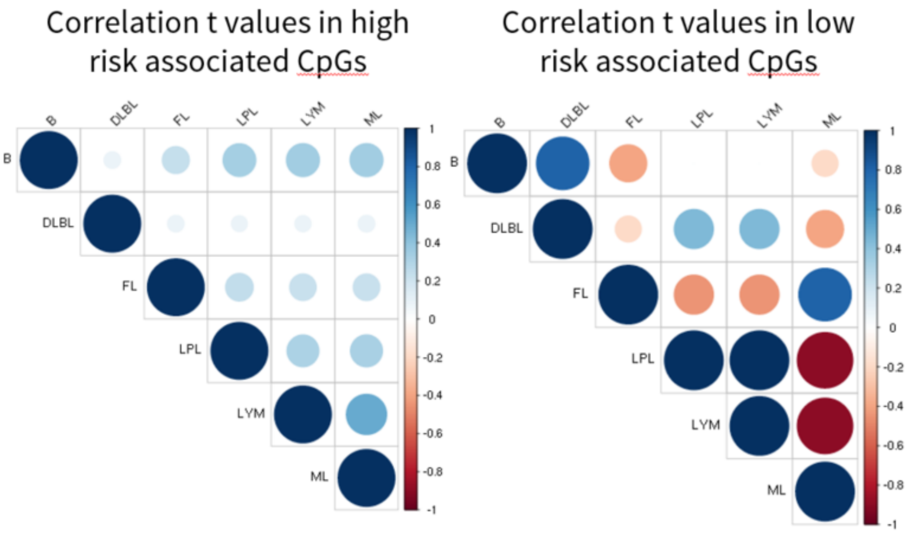

B)

High Risk	B	DLBL	FL	LPL	LYM	ML
B	1.00	0.09	0.23	0.34	0.34	0.34
DLBL	0.09	1.00	0.09	0.08	0.08	0.09
FL	0.23	0.09	1.00	0.24	0.22	0.22
LPL	0.34	0.08	0.24	1.00	0.32	0.32
LYM	0.34	0.08	0.22	0.32	1.00	0.50
ML	0.34	0.09	0.22	0.32	0.50	1.00

Low Risk	B	DLBL	FL	LPL	LYM	ML
B	1.00	0.09	0.23	0.34	0.34	0.34
DLBL	0.09	1.00	0.09	0.08	0.08	0.09
FL	0.23	0.09	1.00	0.24	0.22	0.22
LPL	0.34	0.08	0.24	1.00	0.32	0.32
LYM	0.34	0.08	0.22	0.32	1.00	0.50
ML	0.34	0.09	0.22	0.32	0.50	1.00

Supplementary Figure 8. Top 50 of hyper/hypo-methylated CpGs from the signature from each chromatin state within the prospective cohort (A). Top 50 pathways from GSEA analysis using the C7-immunologic gene sets (B). Reference=Controls.

A)

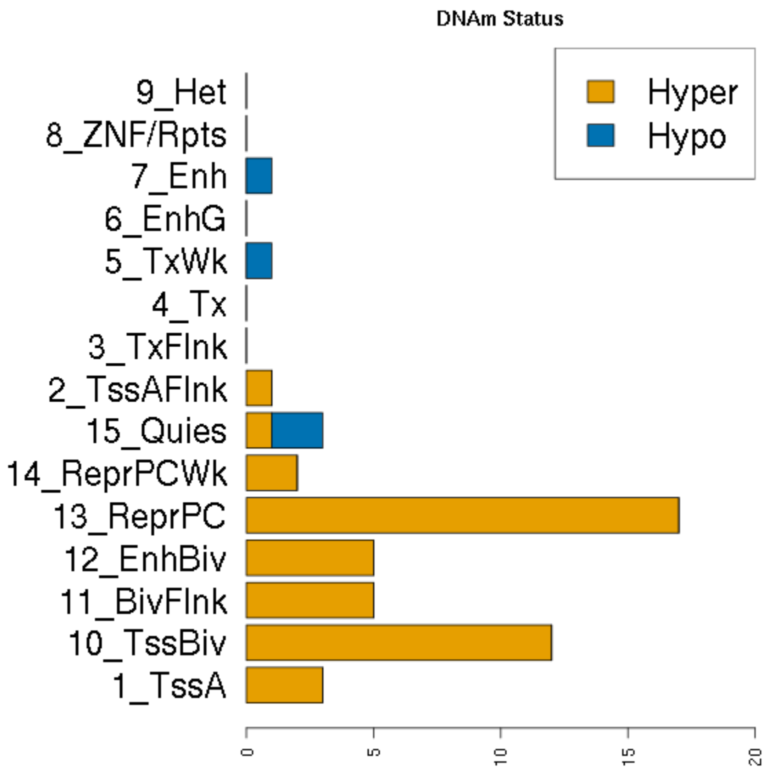

B)

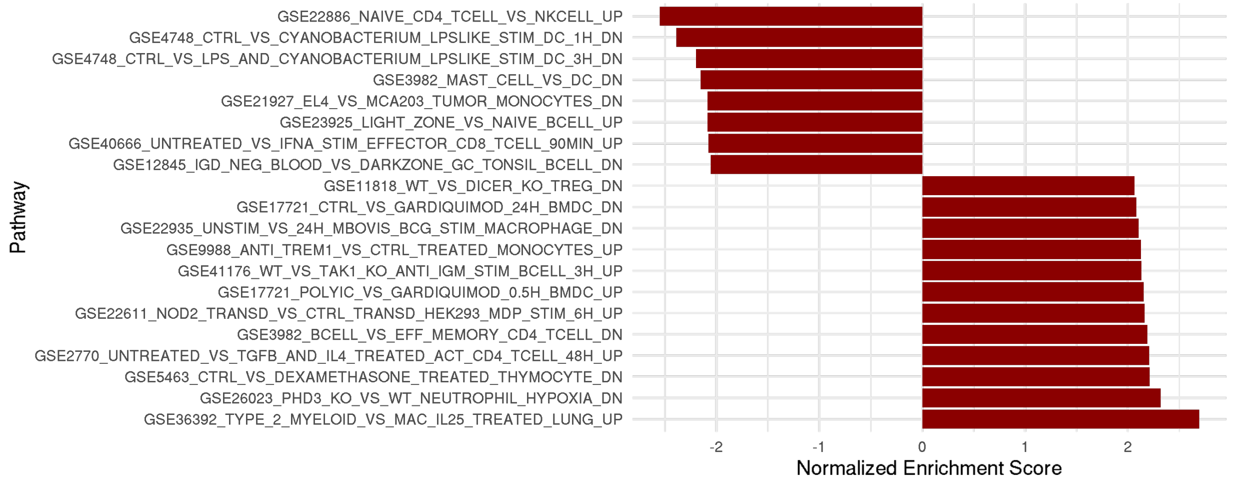

Supplement: Supplementary file 1 — Supplemental Material [file 41698_2022_295_MOESM1_ESM.pdf]
