# Supplementary material for: Peripheral blood DNA methylation profiles predict future development of B-cell Non-Hodgkin Lymphoma
Source: NPJ Precis Oncol. 2022 Jul 21;6:53. doi: 10.1038/s41698-022-00295-3 (PMC9304422; doi:10.1038/s41698-022-00295-3)
Supplement: Supplementary file 5 — REPORTING SUMMARY [file 41698_2022_295_MOESM5_ESM.pdf]

nature portfolio

Corresponding author(s): DBPR-NPJPRECISIONONCOLOGY-01579R

Last updated by author(s): Apr 11, 2022

Reporting Summary

Nature Portfolio wishes to improve the reproducibility of the work that we publish. This form provides structure for consistency and transparency in reporting. For further information on Nature Portfolio policies, see our Editorial Policies and the Editorial Policy Checklist.

Statistics

For all statistical analyses, confirm that the following items are present in the figure legend, table legend, main text, or Methods section.

n/a	Confirmed	
☐	☒	The exact sample size (*n*) for each experimental group/condition, given as a discrete number and unit of measurement
☐	☒	A statement on whether measurements were taken from distinct samples or whether the same sample was measured repeatedly
☐	☒	The statistical test(s) used AND whether they are one- or two-sided<br>*Only common tests should be described solely by name; describe more complex techniques in the Methods section.*
☐	☒	A description of all covariates tested
☐	☒	A description of any assumptions or corrections, such as tests of normality and adjustment for multiple comparisons
☐	☒	A full description of the statistical parameters including central tendency (e.g. means) or other basic estimates (e.g. regression coefficient) AND variation (e.g. standard deviation) or associated estimates of uncertainty (e.g. confidence intervals)
☐	☒	For null hypothesis testing, the test statistic (e.g. *F*, *t*, *r*) with confidence intervals, effect sizes, degrees of freedom and *P* value noted<br>*Give P values as exact values whenever suitable.*
☒	☐	For Bayesian analysis, information on the choice of priors and Markov chain Monte Carlo settings
☒	☐	For hierarchical and complex designs, identification of the appropriate level for tests and full reporting of outcomes
☐	☒	Estimates of effect sizes (e.g. Cohen's *d*, Pearson's *r*), indicating how they were calculated

Our web collection on statistics for biologists contains articles on many of the points above.

Software and code

Policy information about availability of computer code

Data collection	No software was used for data collection
Data analysis	R code. The code implementation is available at https://github.com/alespe/PredDNAm.

For manuscripts utilizing custom algorithms or software that are central to the research but not yet described in published literature, software must be made available to editors and reviewers. We strongly encourage code deposition in a community repository (e.g. GitHub). See the Nature Portfolio guidelines for submitting code & software for further information.

Data

Policy information about availability of data

All manuscripts must include a data availability statement. This statement should provide the following information, where applicable:

- Accession codes, unique identifiers, or web links for publicly available datasets
- A description of any restrictions on data availability
- For clinical datasets or third party data, please ensure that the statement adheres to our policy

The data is available at https://github.com/alespe/PredDNAm. A portion of the dataset is not publicly available due to restrictions imposed by Swedish legislation on the protection of personal data, but is available under request (contact person Ingvar Bergdahl, ingvar.bergdahl@umu.se). The validation datasets are GSE40279, GSE37362, GSE42372, GSE109381 and The Cancer Genome Atlas (TCGA) DLBC (https://www.cancer.gov/tcga).

Field-specific reporting

Please select the one below that is the best fit for your research. If you are not sure, read the appropriate sections before making your selection.

☒ Life sciences ☐ Behavioural & social sciences ☐ Ecological, evolutionary & environmental sciences

For a reference copy of the document with all sections, see nature.com/documents/nr-reporting-summary-flat.pdf

Life sciences study design

All studies must disclose on these points even when the disclosure is negative.

Sample size	After providing written informed consent, blood samples were prospectively collected from subjects who were healthy at enrolment (47,749 volunteers within the EPIC-Italy study and 80,000 subjects within the NSHDS study). Instances of NHL occurring during the study period (16 years) were identified through local Cancer Registries (loss to follow-up<2%).
Data exclusions	For subsequent analyses, we removed B cell chronic lymphocytic leukemia (BCLL) and multiple myeloma (MM), as these are distinct clinical entities from the remaining NHL types, after which 278 samples remained (139 future NHL, and 139 age- and sex-matched controls; Table 1).
Replication	The predictive score was also sensitive at detecting active NHL (96.3% accuracy) and healthy status (95.6% accuracy) in additional independent cohorts.
Randomization	We studied molecular profiles from blood that was collected from apparently healthy adult volunteers who were followed for up to 16 years. From these, 234 were selected who subsequently developed NHL, along with 236 who did not, resulting in a total of 470 samples (Table 1).<br>For each NHL case identified within the two cohorts during follow-up, one random control was selected among all cohort subjects free of cancer at the time of diagnosis, matched by cohort, center, gender, date of blood collection (± 6 months) and age at recruitment (± 2.5 years). Participants diagnosed with disease within less than two years of blood sample collection were excluded.
Blinding	blinding was not relevant to this study, 234 were selected because they developed NHL, along with 236 who did not.

Reporting for specific materials, systems and methods

We require information from authors about some types of materials, experimental systems and methods used in many studies. Here, indicate whether each material, system or method listed is relevant to your study. If you are not sure if a list item applies to your research, read the appropriate section before selecting a response.

Materials & experimental systems

n/a	Involved in the study	
☒	☐	Antibodies
☒	☐	Eukaryotic cell lines
☒	☐	Palaeontology and archaeology
☒	☐	Animals and other organisms
☐	☒	Human research participants
☒	☐	Clinical data
☒	☐	Dual use research of concern

Methods

n/a	Involved in the study	
☒	☐	ChIP-seq
☒	☐	Flow cytometry
☒	☐	MRI-based neuroimaging

Human research participants

Policy information about studies involving human research participants

Population characteristics	We studied DNA methylation from two prospective cohorts where blood was collected from apparently healthy adult volunteers who were followed for up to 16 years. Status disease, sex, age and BMI are described in table 1.
Recruitment	The study population is based on participants from two existing prospective cohorts: the Italian component of the European Prospective Investigation into Cancer and Nutrition (EPIC-Italy) and the Northern Sweden Health and Disease Study (NSHDS). After providing written informed consent, blood samples were prospectively collected from subjects who were healthy at enrolment (47,749 volunteers within the EPIC-Italy study and 80,000 subjects within the NSHDS study). Instances of NHL occurring during the study period (16 years) were identified through local Cancer Registries (loss to follow-up<2%).<br>For each NHL case identified within the two cohorts during follow-up, one random control was selected among all cohort subjects free of cancer at the time of diagnosis, matched by cohort, center, gender, date of blood collection (± 6 months) and age at recruitment (± 2.5 years). Participants diagnosed with disease within less than two years of blood sample collection were excluded.
Ethics oversight	This study was approved by the committee on research ethics at the relevant institutions in accordance with the Declaration

Ethics oversight	of Helsinki of the World Medical Association. All participants provided written informed consent to take part in the study at recruitment

Note that full information on the approval of the study protocol must also be provided in the manuscript.
